# Association Between Aspirin Use and Decreased Risk of Pneumonia in Patients With Cardio-Cerebra-Vascular Ischemic Disease: A Population-Based Cohort Study

**DOI:** 10.3389/fpubh.2021.625834

**Published:** 2021-03-18

**Authors:** Ying-Cheng Chen, Yin-Yang Chen, Han Wei Yeh, Tung-Ying Yeh, Jing-Yang Huang, Pei-Lun Liao, Liang-Tsai Yeh, Shun-Fa Yang, Ming-Chih Chou, Chao-Bin Yeh

**Affiliations:** ^1^Institute of Medicine, Chung Shan Medical University, Taichung, Taiwan; ^2^Department of Surgery, Changhua Christian Hospital, Changhua, Taiwan; ^3^Department of Surgery, Chung Shan Medical University Hospital, Taichung, Taiwan; ^4^School of Medicine, Chang Gung University, Taoyuan City, Taiwan; ^5^Graduate School of Dentistry, School of Dentistry, Chung Shan Medical University, Taichung, Taiwan; ^6^Department of Dentistry, Chung Shan Medical University Hospital, Taichung, Taiwan; ^7^Department of Medical Research, Chung Shan Medical University Hospital, Taichung, Taiwan; ^8^Department of Anesthesiology, Changhua Christian Hospital, Changhua, Taiwan; ^9^Department of Emergency Medicine, School of Medicine, Chung Shan Medical University, Taichung, Taiwan; ^10^Department of Emergency Medicine, Chung Shan Medical University Hospital, Taichung, Taiwan

**Keywords:** aspirin, pneumonia, risk, database, cardio-cerebra-vascular ischemic diseases

## Abstract

This study evaluated the association between long-term low-dose aspirin use and decreased risk of pneumonia in patients with cardio-cerebra-vascular ischemic diseases (CCVDs). This retrospective cohort study used records from Taiwan's National Health Insurance Research Database of claims made between 1997 and 2013. After propensity score matching (PSM), patients who took a low dose of aspirin for more than 90 days within 1 year of diagnosis with CCVDs were identified as the exposure group (*n* = 15,784). A matched total of 15,784 individuals without aspirin use were selected for the non-aspirin group. The main outcome was the development of pneumonia after the index date. Multivariable Cox regression analysis and Kaplan–Meier survival analysis were performed to estimate the adjusted hazard ratio (aHR) and cumulative probability of pneumonia. The result after PSM indicated a lower hazard ratio for pneumonia in aspirin users (aHR = 0.890, 95% confidence interval = 0.837–0.945). Therefore, patients with CCVDs who took aspirin had a lower risk of developing pneumonia than those who did not. In conclusion, this population-based cohort study demonstrated that long-term low-dose aspirin use is associated with a slightly decreased risk of pneumonia in patients with CCVDs.

## Introduction

Cardio-cerebra-vascular ischemic diseases (CCVDs), a class of disorders involving the heart and blood vessels, are the major leading causes of death worldwide, contributing to decreased quality of life and increased economic burden on patients. CCVDs include coronary heart disease, cerebrovascular disease, peripheral arterial disease, rheumatic heart disease, congenital heart disease, deep vein thrombosis, and pulmonary embolism (1). In Taiwan, coronary heart disease and cerebrovascular disease are the 2nd and 4th leading causes of death, respectively, with mortality rates of 48.8 and 26.1% (2). The average medical costs for the 1st year of myocardial infarction, stoke, and angina were reported to be NT$293,995, NT$141,086, and NT$60,305, respectively (3).

Moreover, CCVDs puts patients at higher risk of comorbidities such as hypertension, diabetes mellitus, pneumonia, heart failure, and arrhythmia. Among these, pneumonia is the 3rd leading cause of death in Taiwan (2). A population-based retrospective cohort study reported that patients with CCVDs had a higher risk of pneumonia than did those without [adjusted hazard ratio (aHR): 2.27, 95% confidence interval (CI): 2.01–2.56, *P* < 0.001) (4). The coexistence of CCVDs and pneumonia is associated with a high rate of pneumonia-associated 30-day mortality [hazard ratio (HR), 5.490, CI: 2.905–10.374, *P* < 0.001] (5). Prevention and early treatment of pneumonia in patients with CCVDs is crucial.

Other than lifestyle modifications, antiplatelet therapies such as aspirin play a crucial role for primary and secondary prevention of CCVDs. The cardioprotective effect of aspirin is mainly attributed to the irreversible acetylation of cyclooxygenase (COX)-1 and COX-2 in platelets, resulting in blockade of the production of thromboxane A2 and prostaglandin I2 (PGI2) and inhibiting platelet aggregation and vasoconstriction (6–9). Benefits are achieved with low-dose aspirin (75–100 mg orally daily) according to current trials (10) and guidelines (11).

The anti-infection effects of aspirin have also been studied. The mechanisms by which aspirin affects the immune system and manipulates processes involved in sepsis include the inhibition of COX (12) and nuclear factor kappa B (NF-κB) (13) and the induction of the production of nitric oxide (14) and lipoxin (15). Falcone et al. demonstrated an association between long-term use of aspirin and lower mortality rates (2.07, CI: 1.08–3.98, *P* = 0.029) in patients with pneumonia (16). Other studies have reported protective effects against viral infections of the respiratory tract (17), infectious endocarditis (18), and pyogenic liver abscess (19).

By contrast, Yayan's retrospective cohort study indicated that rather than exhibit significant anti-infection effects in patients with chronic obstructive pulmonary disease (COPD), aspirin use corresponded to increased infection rates (20). Eisen et al. designed the “AspiriN To Inhibit SEPSIS (ANTISEPSIS) randomized controlled trial protocol” for further research into this topic, but the final data have yet to be reported (21). Moreover, McNeil et al. demonstrated increased all-cause mortality in patient with daily low dose aspirin without indication (22). In response to the lack of consensus on the anti-infection effect of aspirin and the high mortality rate of pneumonia in patients with CCVDs, this study evaluated the association between long-term aspirin use and risk of pneumonia in patients with CCVDs in Taiwan. We hypothesized that aspirin can reduce the risk of pneumonia in patients with CCVDs.

## Materials and Methods

### Data Source

We used the Longitudinal Health Insurance Database (LHID) 2000, which is a subset of the National Health Insurance Research Database (NHIRD), to evaluate the effect of aspirin on the risk of pneumonia. The NHIRD provides real-world evidence for exploring the risk factors or effects of an intervention for specific diseases (23). The LHID 2000 consists of the data of one million beneficiaries who were insured by the National Health Insurance program in 2000. The research timeframe in this study was the 17 years between January 1997 and December 2013. This retrospective population-based cohort study was approved by the National Health Insurance Administration and the Institutional Review Board of Chung Shan Medical University (registration number: CSMUH CS16183).

### Study Population

**Figure 1:**
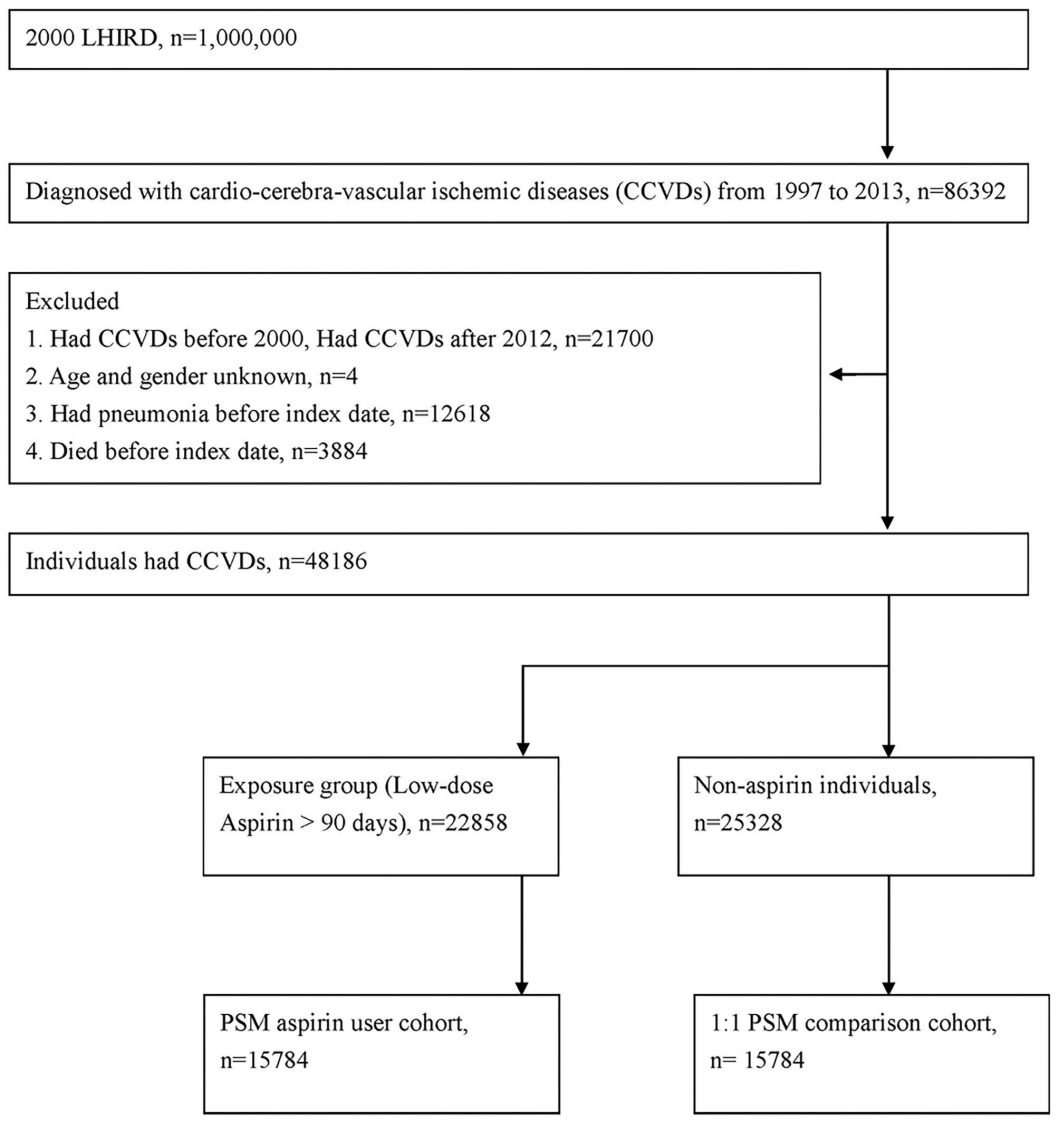
Flowchart of patient selection.

We included patients with a diagnosis of a CCVDs, including coronary artery disease and ischemic stroke (using International Classification of Diseases, Ninth Revision, Clinical Modification [ICD-9-CM] codes 410.x to 414. × and 433. × to 436. ×), between January 1, 1997, and December 31, 2013. To reduce the false positive rate of identifying diseases with ICD-9-CM codes (24), patients were excluded if they had outpatient visit records with CCVDs only. The index date was 365 days after the date of first diagnosis with CCVDs. We excluded patients who (a) had index dates before January 2001 (for left-censored or left-truncated data) or after December 2013 (limited due to the research timeframe), (b) were missing demographic data, (c) died before the index date, or (d) developed pneumonia before the index date. In this study, the censored data including withdraw from insurance coverage, death, and end of study (December 31, 2013). Figure 1 illustrates the study framework.

Initially, 22,858 patients with CCVDs and low-dose aspirin use for more than 90 days after their first diagnosis were classified into the aspirin user group. We evaluated the dose of aspirin within 1 year after diagnosis of CCVDs, the median (Q1, Q3) duration of long-term (100 mg per day) aspirin usage was 275-day (152, 365) and the average medication possession ratio (MPR) was 0.71 in exposure group. By contrast, the median of duration was 1-day (0, 15) with average MPR of 0.03 in non-exposure group within 1 year. In addition, we identified the overall dosage during follow up, the median of duration was 807-day with average MPR of 0.58 in exposure group, and the median of duration was 26-day with average MPR of 0.08 in non-exposure group. The remaining individuals comprised the non-aspirin cohort. To reduce potential confounding bias in our results, 1:1 propensity score matching (PSM) was performed using greedy nearest neighbor, non-replacement matching with a caliper width of 0.01. PSM analysis is widely used in retrospective studies to eliminate imbalance in measured confounding factors among subjects in two study groups. The propensity score of exposure was estimated using logistic regression and covariates, including age, sex, and comorbidities (e.g., hypertension, diabetes mellitus, hyperlipidemia, chronic obstruction pulmonary disease (COPD), dementia, cancer, or major bleeding) and co-medication [including corticosteroids, non-steroidal anti-inflammatory drugs (NSAIDs), proton-pump inhibitors (PPIs), calcium channel blockers (CCBs), Angiotensin-converting-enzyme inhibitors (ACEIs) and statin]. Standardized differences (SDs) were estimated to evaluate the success of balancing baseline covariates between the two study groups; a SD absolute value of <0.1 indicated that the item was balanced between the two groups (25).

### Characteristics, Comorbidities, and Study Outcome

Baseline demographic characteristics, such as age and sex, were recorded. Comorbidities, including hypertension, diabetes mellitus, hyperlipidemia, COPD, dementia, cancer, major bleeding, corticosteroids, NSAIDs, PPIs, CCBs, ACEIs and statin within 1 year before the index date, were documented as potential confounding factors. The study event was defined as the diagnosis of pneumonia (ICD-9-CM 480-486.) during an emergency visit or upon hospital admission. All study individuals were followed up from the index date to the study event, date of death, or end of the study (December 31, 2013).

### Statistical Analysis

Categorical data are presented as numbers and percentages and were compared using a chi-square test. The incidence rate ratio with corresponding CIs and crude HR were calculated using Poisson regression. After the proportional hazard assumption was tested, Cox proportional hazard model analysis was performed to estimate the HR for pneumonia and 95% CI. Statistical analysis was performed using SAS software version 9.4 (SAS Institute, Cary, NC, USA). The significance level was set at 0.05. The cumulative probability of pneumonia was assessed using Kaplan-Meier analysis, in which statistical significance was based on a log-rank test.

## Results

### Characteristics of Study Subjects

We identified 48,186 patients who had received a CCVDs diagnosis during the period from 2000 to 2012. Of these, 22,858 (47.43%) were using a low dose of aspirin for secondary prevention. The aspirin group had a higher proportion of male patients (61.27%), elderly patients, and patients with comorbidities (such as hypertension, diabetes mellitus, and hyperlipidemia); however, a lower proportion of major bleeding was found in the aspirin group. After PSM, a matched aspirin cohort of 15,784 and non-aspirin cohort of the same number were established for analysis. A comparison of the characteristics of the aspirin users and the non-aspirin users is presented in Table 1. Hypertension was the comorbidity of highest prevalence in both groups, followed by hyperlipidemia and diabetes mellitus. Age, gender, comorbidities, and co-medication of corticosteroids, NSAIDs, PPIs, CCBs, ACEIs and statin were not significantly different between the propensity score matched aspirin and non-aspirin groups.

**Table 1:** Baseline characteristics among study groups.

	**Before PSM**			**After PSM**		
**Variable**	**Non-aspirin *N* = 25,328**	**Aspirin *n* = 22,858**	**Standardized difference**	**Non-aspirin *N* = 15,784**	**Aspirin *n* = 15,784**	**Standardized difference**
**Sex**			0.17834			0.03814
Female	12,039 (47.53%)	8,854 (38.73%)		6,943 (43.99%)	6,645 (42.1%)	
Male	13,289 (52.47%)	14,004 (61.27%)		8,841 (56.01%)	9,139 (57.9%)	
**Age**			0.40063			0.09334
≤ 50	6,136 (24.23%)	2,306 (10.09%)		1,806 (11.44%)	1,754 (11.11%)	
51–60	5,325 (21.02%)	4,768 (20.86%)		3,123 (19.79%)	3,340 (21.16%)	
61–70	5,458 (21.55%)	6,387 (27.94%)		4,001 (25.35%)	4,335 (27.46%)	
71–80	5,583 (22.04%)	6,755 (29.55%)		4,572 (28.97%)	4,581 (29.02%)	
≥81	2,826 (11.16%)	2,642 (11.56%)		2,282 (14.46%)	1,774 (11.24%)	
**Comorbidities**						
Hypertension	16,044 (63.34%)	19,573 (85.63%)	0.52873	13,312 (84.34%)	12,857 (81.46%)	−0.07661
Diabetes mellitus	7,096 (28.02%)	9,503 (41.57%)	0.28756	5,867 (37.17%)	5,831 (36.94%)	−0.00472
Hyperlipidemia	7,944 (31.36%)	12,335 (53.96%)	0.46934	6,740 (42.7%)	6,836 (43.31%)	0.01229
COPD	5,983 (23.62%)	5,515 (24.13%)	0.01185	4,113 (26.06%)	3,993 (25.3%)	−0.01740
Dementia	1,596 (6.30%)	1,690 (7.39%)	0.04325	1,216 (7.7%)	1,208 (7.65%)	−0.00190
Cancer	2,071 (8.18%)	1,673 (7.32%)	−0.03208	1,292 (8.19%)	1,240 (7.86%)	−0.01213
Major bleeding	5,970 (23.57%)	4,427 (19.37%)	−0.10250	3,557 (22.54%)	3,538 (22.42%)	−0.00288
**Medication**						
corticosteroids	12,963 (51.18%)	11,273 (49.32%)	−0.03727	8,147 (51.62%)	7,981 (50.56%)	−0.02104
NSAIDs	23,370 (92.27%)	20,814 (91.06%)	−0.04384	14,516 (91.97%)	14,476 (91.71%)	−0.00926
PPIs	5,329 (21.04%)	3,714 (16.25%)	−0.12327	3,128 (19.82%)	2,979 (18.87%)	−0.02390
CCBs	15,089 (59.57%)	17,737 (77.6%)	0.39579	12,073 (76.49%)	11,705 (74.16%)	−0.05410
ACEIs	8,277 (32.68%)	12,088 (52.88%)	0.41714	7,378 (46.74%)	7,368 (46.68%)	−0.00127
Statin	5,205 (20.55%)	10,906 (47.71%)	0.59790	5,015 (31.77%)	5,389 (34.14%)	0.05042

*COPD, Chronic Obstruction Pulmonary Disease; NSAIDs, Non-steroidal anti-inflammatory drugs; PPIs, Proton-pump inhibitors; CCBs, Calcium channel blockers; ACEIs, Angiotensin-converting-enzyme inhibitors*.

### Risk of Pneumonia for Different Comorbidities and Other Conditions

**Figure 2:**
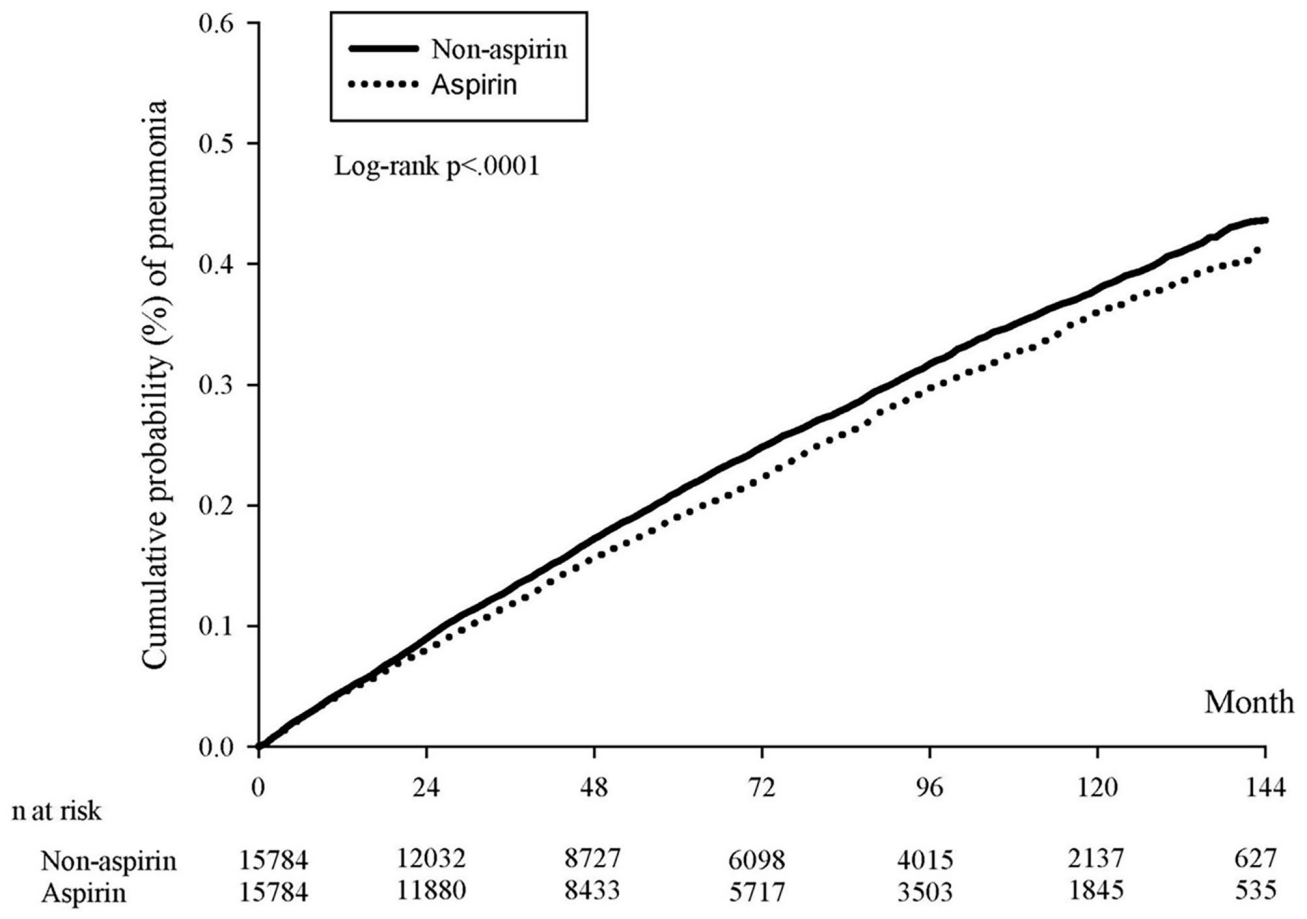
Kaplan–Meier curves of the cumulative proportions of pneumonia after propensity score matching.

After PSM, the incidence densities of pneumonia (per 1,000 person months) were 3.60 (CI = 3.48–3.73) and 3.95 (CI = 3.82–4.07) in the aspirin and non-aspirin cohorts, respectively. A Kaplan–Meier survival analysis revealed a significantly lower cumulative incidence of pneumonia in the aspirin group (Figure 2). Compared with patients in the non-aspirin group, those with CVD in the aspirin group exhibited a decreased risk of pneumonia (aHR: 0.890, CI: 0.837–0.945). Other significant risk factors of pneumonia in patients with CCVDs included dementia, DM and COPD (Table 2). The stratified analysis showed the significant protect effect of aspirin in subgroup of male, hypertension, non-DM, COPD, non-dementia, non-cancer, and patients without major bleeding (Table 3).

**Table 2:** Multiple Cox proportional hazard regression results for pneumonia.

	**Before PSM**		**After PSM**	
**Variable**	**aHR (95% C.I.)**	***P*-value**	**aHR (95% C.I.)**	***P*-value**
**Aspirin**				
No	Reference		Reference	
Yes	0.952 (0.913–0.992)	0.0199	0.890 (0.837–0.945)	0.0002
**Sex**				
Female	Reference			
Male	1.351 (1.297–1.408)	<0.0001		
**Age**				
≤ 50	Reference			
51–60	1.437 (1.303–1.584)	<0.0001		
61–70	2.328 (2.129–2.546)	<0.0001		
71–80	4.184 (3.837–4.563)	<0.0001		
≥81	7.717 (7.037–8.463)	<0.0001		
**Comorbidities (ref: non)**				
Hypertension	1.034 (0.973–1.098)	0.2851		
Diabetes mellitus	1.436 (1.377–1.496)	<0.0001		
Hyperlipidemia	0.847 (0.806–0.891)	<0.0001		
COPD	1.363 (1.307–1.422)	<0.0001		
Dementia	1.929 (1.816–2.048)	<0.0001		
Cancer	1.225 (1.147–1.309)	<0.0001		
Major bleeding	1.210 (1.155–1.268)	<0.0001		
**Medication**				
Corticosteroids	1.134 (1.089–1.181)	<0.0001		
NSAIDs	0.943 (0.874–1.017)	0.1297		
PPIs	1.127 (1.070–1.186)	<0.0001		
CCBs	1.133 (1.073–1.196)	<0.0001		
ACEIs	1.150 (1.102–1.199)	<0.0001		
Statin	0.962 (0.912–1.016)	0.1641		

* *aHR, adjusted hazard ratio; COPD, Chronic Obstruction Pulmonary Disease; NSAIDs, Non-steroidal anti-inflammatory drugs; PPIs, Proton-pump inhibitors; CCBs, Calcium channel blockers; ACEIs, Angiotensin-converting-enzyme inhibitors*.

**Table 3:** Risk of pneumonia among aspirin and non-aspirin groups and stratified analysis.

**Variables**	**Incidence rate**		**Adjusted HR^†^**	**95% CI**	
	**Non-aspirin**	**Aspirin**		**Lower**	**Upper**
Study groups	3.95 (3.82–4.07)	3.60 (3.48–3.73)	0.890^**^	0.837	0.945
**Sex**					
Female	3.39 (3.22–3.57)	3.46 (3.28–3.64)	1.016	0.911	1.132
Male	4.41 (4.23–4.59)	3.71 (3.55–3.88)	0.802^***^	0.735	0.876
**Age**					
<70	2.18 (2.06–2.30)	1.99 (1.88–2.11)	0.824^**^	0.732	0.928
≥70	6.65 (6.39–6.91)	6.30 (6.04–6.57)	0.981	0.897	1.072
**Comorbidities**					
**Hypertension**					
Without	3.04 (2.79–3.32)	2.72 (2.49–2.97)	0.932	0.760	1.143
With	4.12 (3.99–4.27)	3.81 (3.68–3.95)	0.892^**^	0.834	0.954
**Diabetes mellitus**					
Without	3.58 (3.43–3.73)	3.22 (3.08–3.37)	0.896^*^	0.822	0.978
With	4.63 (4.41–4.86)	4.31 (4.09–4.54)	0.910	0.815	1.015
**Hyperlipidemia**					
Without	4.58 (4.40–4.76)	4.13 (3.96–4.31)	0.924	0.851	1.003
With	3.11 (2.94–3.28)	2.89 (2.73–3.06)	0.915	0.813	1.028
**COPD**					
Without	3.25 (3.12–3.38)	3.00 (2.88–3.13)	0.889^**^	0.818	0.965
With	6.03 (5.73–6.35)	5.45 (5.15–5.76)	0.837^**^	0.743	0.944
**Dementia**					
Without	3.65 (3.53–3.77)	3.25 (3.13–3.37)	0.861^***^	0.805	0.921
With	9.18 (8.40–10.0)	10.1 (9.32–11.1)	1.127	0.863	1.472
**Cancer**					
Without	3.80 (3.68–3.93)	3.46 (3.34–3.58)	0.897^**^	0.840	0.958
With	6.04 (5.47–6.68)	5.73 (5.15–6.37)	1.066	0.779	1.457
**Major bleeding**					
Without	3.63 (3.50–3.77)	3.29 (3.16–3.42)	0.874^**^	0.811	0.941
With	5.18 (4.87–5.51)	4.86 (4.55–5.19)	0.969	0.841	1.117
**Medication**					
**Corticosteroids**					
Without	3.51 (3.35–3.69)	3.20 (3.04–3.36)	0.831^**^	0.748	0.923
With	4.36 (4.18–4.54)	4.00 (3.83–4.19)	0.963	0.879	1.055
**NSAIDs**					
Without	3.79 (3.38–4.26)	3.16 (2.79–3.59)	0.897	0.635	1.268
With	3.96 (3.83–4.09)	3.64 (3.52–3.77)	0.897^**^	0.841	0.957
**PPIs**					
Without	3.78 (3.65–3.92)	3.39 (3.26–3.52)	0.861^***^	0.802	0.924
With	4.81 (4.48–5.17)	4.82 (4.47–5.19)	1.114	0.938	1.324
**CCBs**					
Without	3.13 (2.91–3.37)	2.91 (2.70–3.14)	0.891	0.751	1.059
With	4.19 (4.05–4.34)	3.83 (3.69–3.98)	0.905^**^	0.843	0.972
**ACEIs**					
Without	3.40 (3.24–3.56)	3.38 (3.22–3.55)	0.973	0.879	1.076
With	4.54 (4.35–4.74)	3.83 (3.66–4.02)	0.842^**^	0.767	0.923
**Statin**					
Without	4.20 (4.05–4.35)	3.99 (3.84–4.15)	0.924^*^	0.858	0.994
With	3.32 (3.11–3.54)	2.72 (2.53–2.92)	0.844^*^	0.734	0.969

* *p < 0.05*,

** *p < 0.01*,

*** *p < 0.0001*;

† *Adjusted for all variables*.

*COPD, Chronic Obstruction Pulmonary Disease; NSAIDs, Non-steroidal anti-inflammatory drugs; PPIs, Proton-pump inhibitors; CCBs, Calcium channel blockers; ACEIs, Angiotensin-converting-enzyme inhibitors*.

## Discussion

This is the first nationwide longitudinal population-based cohort study to evaluate the effect of aspirin on pneumonia in patients with CCVDs, and the findings indicate that long-term low-dose aspirin use is associated with a slightly decreased risk of pneumonia. Aspirin is well-known for secondary prevention in patients with CCVDs and reduces the mortality and disease recurrence of CCVDs (10, 11). We discovered its further ability to reduce the incidence of pneumonia in patients with CCVDs. Nevertheless, for patients with hemorrhagic stroke, how aspirin use affects the risk of major bleeding is still under debate. Hence, we only included patients with ischemic stroke in this study population.

Our findings are consistent with those of other studies on the association between aspirin and infection. A literature review revealed the ability of aspirin to prevent infections. Eisen et al. performed a retrospective cohort study in an intensive care unit setting that showed that the administration of aspirin was associated with 14.8% lower mortality in critically ill patients with sepsis (26). Another nationwide population-based cohort study published in 2015 reported that the administration of aspirin was associated with survival benefit in patients with sepsis (27). Furthermore, a prospective cohort study published in the same year demonstrated that elderly patients with pneumonia who received a low daily dose of aspirin (100 mg/day) had a lower total mortality rate than did those who did not receive any medication (16). In our population, 47.43% of patients were treated with long-term low-dose aspirin and were predominantly male, of old age, and with comorbidities of hypertension, diabetes mellitus, or hyperlipidemia and use of PPI before PSM.

The mechanisms by which aspirin affects the immune system has three main pathways, involving tumor necrosis factor (TNF), lipid mediators, and platelets (21). First, receptors of immune cells can recognize pathogen-associated molecular patterns and modulate intracellular signaling, resulting in the activation of NFκB and the transcription of TNF, a proinflammatory cytokine. Aspirin regulates immune response by inhibiting the activation of NFκB (28). Second, lipid mediators play a part in anti-inflammation and the restoration of homeostasis (29). Low-dose aspirin use increases the number of lipid mediators of lipoxins and resolvins, which inhibit the production of proinflammatory cytokines (30). Finally, aspirin inhibits the activation and aggregation of platelets, and its role in the interaction between platelets and pathogens in immune responses and infections has recently been researched widely (31, 32). The surface receptors of platelets are involved in direct platelet–bacteria interactions, and plasma proteins promote indirect interactions, resulting in the modulation of neutrophils, Kupffer cells, and the complement system (33).

In regard to the definition of pneumonia in our study was described as follows. According to our study design, the index date was 365 days after the date of first diagnosis of CCVDs and patients with previous pneumonia diagnosis were excluded and we only included the newly diagnosis of pneumonia with ICD 9 code from emergencies or hospitalization to confirm the accuracy of diagnosis. Hence, if the diagnosis of pneumonia was made in ER or upon admission, it should represent for community-acquired pneumonia (CAP). On the contrast, if the diagnosis of pneumonia was made during the hospitalization, it could be hospital-acquired pneumonia (HAP). We could not distinguish CAP from HAP only using ICD 9 code and this is one limitation of NHIRD. However, CAP should be the majority of the diagnosis based on the clinical condition. Hence, our study included all cause of pneumonia instead of CAP only. On the other hand, for the accuracy of using ICD 9 code to claim the data of pneumonia, there is one research on this question: International classification of diseases codes showed modest sensitivity for detecting community-acquired pneumonia. Previous study concluded that ICD-9-CM codes showed modest sensitivity for detecting CAP in hospital administrative databases, leaving at least one quarter of pneumonia cases undetected (34). Therefore, sensitivity decreased with longer duration of hospital stay.

Our study demonstrated a 2-fold risk of pneumonia in patients with COPD and patients with dementia, which is in agreement with the findings of other studies. COPD was associated with a higher risk of pneumonia and an increased mortality rate (35, 36). A recent COPD cohort study showed an incidence rate of pneumonia of 22.4 (CI: 21.7–23.2) per 1,000 person years (37). Furthermore, a single center study in Taiwan demonstrated that COPD patients with CCVDs had an increased risk of pneumonia (38). However, dementia was proven to be a risk factor of pneumonia because of prolonged latency of the swallowing reflex (39). A systemic review and meta-analysis published in 2019 reported that the rate of pneumonia-associated mortality was doubled in patients with dementia compared with those without (40). Patients with COPD and dementia require adequate chest care and cautious clinical management for pneumonia prevention and improved prognoses. The non-protection effect of aspirin in COPD patients with pneumonia was consistent with a previous study mentioned, showing that aspirin was associated with higher COPD acute exacerbation and infection events (20). On the other hand, the non-protection effect of aspirin on diabetes mellitus, dementia and cancer population found in our study needs more research to confirm the relevance. The strengths and novelties of our study are as follows. First, a large population cohort with application of PSM provided a balance of selected covariates. Second, pneumonia is associated with higher mortality in CCVDs patients. Our results demonstrate the clinical benefits of aspirin for pneumonia prevention. Finally, aspirin prescriptions are made for patients with CCVDs after careful consideration of two crucial aspects: the reduced risk of death of CCVDs and the increased risk of major bleeding. The results of our study provide supporting evidence of the reduced risk of pneumonia resulting from aspirin use in patients with CCVDs.

This study had some limitations. First, the NHIRD did not contain information on the severity of CCVDs, performance status, or patients' clinical characteristics such as obesity or smoking status. Second, covariates in this study lacked information on the initial treatment of CCVDs, such as medication usage or the necessity of therapeutic or surgical intervention. Third, data on influenza and pneumococcal vaccinations was not included in this study. In Taiwan, the health policy provided annual free influenza and pneumococcal vaccination since 2001 and 2007, respectively, but with age limitation. Annual influenza vaccination was only free for people aged more than 65 years, and pneumococcal vaccination people only for aged more than 75 years, both elder and susceptible group of general population. Finally, a lack of randomization due to the observational nature of this study was an intrinsic limitation. Carefully designed and planned studies using randomized control trials are warranted for precise analysis of the benefits of aspirin.

## Summary

In summary, this population-based cohort study with PSM demonstrated that long-term low-dose aspirin use is associated with a decreased risk of pneumonia in patients with CCVDs, whereas Diabetes mellitus, COPD and dementia are associated with greater risk of pneumonia. Further randomized clinical trials are required to support these findings before strategies for increasing prevalence of aspirin use among patients with CCVDs can be developed.

## Data Availability Statement

The original contributions presented in the study are included in the article/supplementary material, further inquiries can be directed to the corresponding author/s.

## Ethics Statement

This retrospective population-based cohort study was approved by the National Health Insurance Administration and the Institutional Review Board of Chung Shan Medical University (registration number: CSMUH CS16183). Written informed consent for participation was not required for this study in accordance with the national legislation and the institutional requirements.

## Author Contributions

Y-CC, Y-YC, M-CC, and C-BY conceived and designed the experiments. HY, T-YY, J-YH, and P-LL analyzed the data. L-TY, S-FY, and C-BY contributed reagents, materials, and analysis tools. Y-CC, Y-YC, M-CC, and C-BY wrote the paper. All authors contributed to the article and approved the submitted version.

## Conflict of Interest

The authors declare that the research was conducted in the absence of any commercial or financial relationships that could be construed as a potential conflict of interest.

## Abbreviations

aHRs: adjusted hazard ratios
CCVDs: cardio-cerebra-vascular ischemic disease
NHIRD: National Health Insurance Research Database
COPD: Chronic Obstruction Pulmonary Disease
NSAIDs: Non-steroidal anti-inflammatory drugs
PPIs: Proton-pump inhibitors
CCBs: Calcium channel blockers
ACEIs: Angiotensin-converting-enzyme inhibitors.
